# Supplementary material for: The Characteristics and Motivations of Taiwanese People toward Advance Care Planning in Outpatient Clinics at a Community Hospital
Source: Int J Environ Res Public Health. 2021 Mar 10;18(6):2821. doi: 10.3390/ijerph18062821 (PMC7999986; doi:10.3390/ijerph18062821)
Supplement: Supplementary file 1 [file ijerph-18-02821-s001.pdf]

Supplementary materials

Supplementary 1: Definitions of the terms in the study (page 2)

Supplementary 2**:** Advance decision document (English version) (page 3)

Supplementary 3**:** Advance Care Planning Service-Case Report Form (English version) (page 18)

Supplementary 4: Advance Care Planning Service-Service Satisfaction Form (English version) (page 19)

Supplementary 1: Definitions of the terms in the study

Term	Abbreviations	Definition
Advance decisions	ADs	The formal and authorized documents provided by the Ministry of Health and Welfare (MOHW), Taiwan for deciding the preferred medical directives under presumed health conditions.
Advance care planning	ACP	Processes or sessions that facilitate the discussion and completion of ADs by specialized trained medical personnel.
Participant	-	A volunteer individual, either healthy or having underlying illnesses, who participated the full process of ACP consultations with the aim to complete personal ADs.
Healthcare surrogate or healthcare agent	HS or HCA	The assigned third-party individual who will be substituted for future medical care decisions when the subject is unable to express his or her own preferences.
Major illness	-	Catastrophic, severe or debilitating illnesses that are certified and annotated by the National Health Insurance of Taiwan, such as malignancy, end-stage renal disease, or autoimmune disorders.
Life sustaining treatment	LST	Cardiopulmonary resuscitation, electrical cardiac defibrillation, endotracheal intubation, hemodialysis, hemoperfusion, extracorporeal life supports, antimicrobial therapies, and clinically-indicated blood component transfusions or plasma exchange.
Artificial nutrition and/or hydration	ANH	Intravenous hydration or total/partial parenteral nutrition via peripheral or central venous routes and tube feeding via nasogastric, naso-intestinal or gastrostomy routes.

Supplementary 2: Advance decision document (English version)

Advance Care Decisions

I, ______________, after the advance care planning consultation, clearly understand that the *Patient Rights to Autonomy Act* empowers a patient to accept or refuse life-sustaining treatments and artificial nutrition and/or hydration when he/she is under specific clinical conditions. I pledge the advance decisions (as specified in part I, part II and the supplement) to state the end-of-life care I wish to receive and demand the related personnel to respect my will.

Name of declarant:<br>National identification number/ passport number:<br>Address:<br>Phone numbers:<br>Signature:<br>Date: Time:

Witness/Notarization

I choose to make my advance decision legal in the following procedure (please choose one):<br>☐ In the presence of two witnesses<br><br>Witness one:<br>Witness two:<br>☐ In the presence of a notary public service<br><br>Signature of notary public service: Stamp:

Note:

1. A witness must have full disposing capacity and acknowledge that signing of the documents is done voluntarily without coercion (subparagraph 2 of Paragraph 1 of Article 9 of *Patient Right to Autonomy Act*).

2. The following persons may not serve as a witness: the declarant's health care agent, members of the responsible medical team, the declarant's legatee apart from his or her heirs, legatees of the declarant's remains or organs, other persons who shall benefit from the death of the declarant.

3. In accordance with Article 2 of *Notarization Law*, notaries are able to authenticate the juristic acts and facts related to the private rights and approve the documents. Notaries may also attest the following documents upon application of directly related parties or other indirectly related persons:

a. Official documents in their original form that involve facts related to private rights and where the applicants indicate the documents will be used overseas.

b. The transcription or photocopies of official or private documents.

Part I Treatment preferences

Clinical condition 1: Terminal illnesses

A. Life-sustaining treatment

My wish and decision is as follows (please choose one):

- ☐ I don't wish to receive life-sustaining treatments.
- ☐ I wish to try life-sustaining treatments for (please specify a period of time)________________, and to stop them thereafter if they do not help. However, I (the patient) or my health care agent has the power to withdraw life-sustaining treatments at any time during that period.
- ☐ If I am unconscious or unable to express my wishes, my healthcare surrogate can make decisions on my behalf.
- ☐ I wish to receive life-sustaining treatments.

B. Artificial nutrition and/or hydration

My wish and decision is as follows (please choose one):

- ☐ I don't wish to receive artificial nutrition or hydration.

- ☐ I wish to try artificial nutrition and hydration for (please specify a period of time) ______________, and to stop it thereafter if it does not help. However, I (the patient) or my health care agent has the power to withdraw artificial nutrition and hydration at any time during that period.
- ☐ If I am unconscious or unable to express my wishes, my healthcare surrogate can make decisions on my behalf.
- ☐ I wish to receive artificial nutrition and hydration.

Clinical condition 2: Irreversible comatose status

A. Life-sustaining treatment

My wish and decision are as follows (please choose one):
☐ I don't wish to receive life-sustaining treatments.
☐ I wish to try life-sustaining treatments for (please specify a period of time) _______________, and to stop them thereafter if they do not help. However, I (the patient) or my health care agent has the power to withdraw life-sustaining treatments at any time during that period.
☐ If I am unconscious or unable to express my wishes, my healthcare surrogate can make decisions on my behalf.
☐ I wish to receive life-sustaining treatments.

B. Artificial nutrition and/or hydration

My wish and decision are as follows (please choose one):
☐ I don't wish to receive artificial nutrition or hydration.
☐ I wish to try artificial nutrition and hydration for (please specify a period of time) _______________, and to stop it

thereafter if it does not help. However, I (the patient) or my health care agent has the power to withdraw artificial nutrition and hydration at any time during that period.

- ☐ If I am unconscious or unable to express my wishes, my healthcare surrogate can make decisions on my behalf.
- ☐ I wish to receive artificial nutrition and hydration.

Clinical condition 3: Sustained vegetative status

A. Life-sustaining treatment

My wish and decision are as follows (please choose one):

- ☐ I don't wish to receive life-sustaining treatments.
- ☐ I wish to try life-sustaining treatments for (please specify a period of time) ______________, and to stop them thereafter if they do not help. However, I (the patient) or my health care agent has the power to withdraw life-sustaining treatments at any time during that period.
- ☐ If I am unconscious or unable to express my wishes, my healthcare surrogate can make decisions on my behalf.
- ☐ I wish to receive life-sustaining treatments.

B. Artificial nutrition and/or hydration

My wish and decision are as follows (please choose one):

- ☐ I don't wish to receive artificial nutrition or hydration.
- ☐ I wish to try artificial nutrition and hydration for (please specify a period of time) ______________, and to stop it

thereafter if it does not help. However, I (the patient) or my health care agent has the power to withdraw artificial nutrition and hydration at any time during that period.

- ☐ If I am unconscious or unable to express my wishes, my healthcare surrogate can make decisions on my behalf.
- ☐ I wish to receive artificial nutrition and hydration.

Clinical condition 4: Severe debilitating dementia

A. Life-sustaining treatment

My wish and decision are as follows (please choose one):

☐ I don't wish to receive life-sustaining treatments.

☐ I wish to try life-sustaining treatments for (please specify a period of time) ______________, and to stop them thereafter if they do not help. However, I (the patient) or my health care agent has the power to withdraw life-sustaining treatments at any time during that period.

☐ If I am unconscious or unable to express my wishes, my healthcare surrogate can make decisions on my behalf.

☐ I wish to receive life-sustaining treatments.

B. Artificial nutrition and/or hydration

My wish and decision are as follows (please choose one):

☐ I don't wish to receive artificial nutrition or hydration.

☐ I wish to try artificial nutrition and hydration for (please specify a period of time) ______________, and to stop it

thereafter if it does not help. However, I (the patient) or my health care agent has the power to withdraw artificial nutrition and hydration at any time during that period.

- ☐ If I am unconscious or unable to express my wishes, my healthcare surrogate can make decisions on my behalf.
- ☐ I wish to receive artificial nutrition and hydration.

Clinical condition 5: Other unspecified agonizing illnesses

A. Life-sustaining treatment

My wish and decision are as follows (please choose one):

- ☐ I don't wish to receive life-sustaining treatments.

- ☐ I wish to try life-sustaining treatments for (please specify a period of time) ______________, and to stop them thereafter if they do not help. However, I (the patient) or my health care agent has the power to withdraw life-sustaining treatments at any time during that period.

- ☐ If I am unconscious or unable to express my wishes, my healthcare surrogate can make decisions on my behalf.

- ☐ I wish to receive life-sustaining treatments.

B. Artificial nutrition and/or hydration

My wish and decision are as follows (please choose one):

- ☐ I don't wish to receive artificial nutrition or hydration.

- ☐ I wish to try artificial nutrition and hydration for (please specify a period of time) ______________, and to stop it

thereafter if it does not help. However, I (the patient) or my health care agent has the power to withdraw artificial nutrition and hydration at any time during that period.

- ☐ If I am unconscious or unable to express my wishes, my healthcare surrogate can make decisions on my behalf.
- ☐ I wish to receive artificial nutrition and hydration.

Part II Certificate by the medical institution providing consultation on advance care planning

It is certified that (declarant's name)_________________, has consulted with medical service providers about advance care planning on _(date)__________.

Date:

Institution:

(Affix seal)

Appointment of health care agent

I,____________, hereby authorize ________________ to act on my behalf to exercise the rights as listed in Paragraph 3 of Article 10 of *Patient Rights to Autonomy Act.*

(order of precedence:___)

Name of authorized person:<br>National identification number/ passport number:<br>Date of birth:<br>Address:<br>Phone numbers:<br>Signature:<br>Date:

Articles regarding health care agent in the *Patient Rights to Autonomy Act*

Article 10
The health care agent designated by the declarant must be aged 20 years or older, possess full disposing capacity, and must consent to the designation in writing.

Apart from the declarant's heirs, the following persons may not serve as health care agents:
1. The declarant's legatees.
2. Legatees of the declarant's remains or organs.
3. Other persons who shall benefit from the death of the declarant.

When the declarant is unconscious or unable to clearly express his or her wishes, the health care agent may exercise the following rights on behalf of the declarant:
1. Receiving the information set forth in Article 5.
2. Signing the consent form as set forth in Article 6.
3. Expressing the patient's wishes on his or her behalf in accordance with the contents of the patient's advance decision.

When there are more than two health care agents, each of them may represent the declarant independently.

When handling the entrusted matters, the health care agent must provide identity documents to the medical institution or physician.

Article 11
A health care agent may terminate the designation at any time in writing. A health care agent shall, ipso facto, be dismissed under any of the following circumstances:
1. If the health care agent becomes mentally impaired due to a disease or an accident after a relevant medical or psychiatric assessment.
2. If the health care agent becomes subject to the adjudication of the commencement of assistance or guardianship.

Article 13
The declarant must file an application with the central competent authority to renew the registration in the event of any of the following circumstances:
1. Withdrawal or modification of the advance decision.
2. Designation, dismissal, or changing of a health care agent.

Supplementary 3: Advance Care Planning Service-Case Report Form (English version)

Advance Care Planning Service
Case Report Form

Note: the following case report form is to record the basic information of the participant who engages advance care planning (ACP) consultation by the case manager, study nurse or study social worker BEFORE the formal ACP session initiates.

The form is de-identified to individual private information

Basic information:

“We would kindly record some of your basic information to facilitate the following session. If some of the contents are not available, you can choose not to provide them to us.”

The participant (Serial Number: □□□□□□□□□□□)

Age: ____years

Sex: □Male □Female

Residential location:

□Douliou city □other cities in Taiwan_______ □other countries_________

Religion background:

□Buddhism □Taoism □Christian □Catholic □Islam □Atheism □Others_________

Education background (highest):

□Primary school (6-9 grades) □Secondary school (10-12 grades) □College

□Above college □None □Others_________

Marital status:

□Married □Cohabitated □Single □Others_________

Motivations for participation:

“What is/are the motivation/condition/idea for you to participate this consultation today? You can freely choose multiple answers if you want to.”

(either single or multiple choices are allowed)

□ I used to have experiences of passing or caring family members.

□ I have pre-existing medical illnesses.

□ I want to reduce the burden of my family/friend.

□ I am singled or widowed.

□ I want to assign a healthcare surrogate.

□ I want to prepare my life wishes in advance.

□ I believe that the ACP service corresponds with my needs.

□ I am attracted by the propagandas of the service provider (hospitals or clinics).

□ I intend not to waste medical resources when I am in terminal condition.

□ Others, please specify_______________________________

Supplementary 4: Advance Care Planning Service-Service Satisfaction Form (English version)

Advance Care Planning Service
Service Satisfaction Form

Note: the following satisfaction form is to record the subjective satisfactory outcome of the participant who engages advance care planning (ACP) consultation by him/herself RIGHT AFTER the formal ACP session is completed.

The form is de-identified to individual private information

Satisfactory outcome:

"We would kindly invite you to choose your satisfactory grading of our service. Your personal information, service course or content of ADs is unaffected and unrelated by the following results."

1. In general, I feel the ACP consultation today is
□Very good □Good □Fair □Poor □Very poor

2. I feel that my understanding of ACP after the consultation today is
□Very good □Good □Fair □Poor □Very poor

3. I feel that the service respects and supports my end-of-life wishes
□Totally agreed □Agreed □Fair □Disagreed □Totally disagreed

4. I feel the time length or schedule of the session today is
□Very appropriate □Appropriate □Fair □Inappropriate □Very inappropriate

5. I feel the environment or setting of the clinic is
□Very good □Good □Fair □Poor □Very poor

6. I feel the additional service charge is
□Very appropriate □Appropriate □Fair □Inappropriate □Very inappropriate

7. If you have any remarks or comments to share with us,
____

"Thank you for the participation of the service and satisfactory evaluation. Your feedback will provide important information to improve our work and help more future participants."
